# Supplementary material for: Hot spot prediction in protein-protein interactions by an ensemble system
Source: BMC Syst Biol. 2018 Dec 31;12(Suppl 9):132. doi: 10.1186/s12918-018-0665-8 (PMC6311905; doi:10.1186/s12918-018-0665-8)
Supplement: Supplementary file 2 — All relevant MATLAB code. A simple Matlab implement of our predictor. (ZIP 19,742 kb) [file 12918_2018_665_MOESM2_ESM.zip › Additional file 2/Read ASA/ASA website/dbMPIKT/4OFYD.html]

 NetSurfP - Protein Surface Accessibility and Secondary Structure Predictions

  

|  |  |  |
| --- | --- | --- |
|  |  | NetSurfP - Protein Surface Accessibility and Secondary Structure Predictions  Technical University of Denmark |

  


---

```
# For publication of results, please cite:
# A generic method for assignment of reliability scores applied to solvent accessibility predictions.
# Bent Petersen, Thomas Nordahl Petersen, Pernille Andersen, Morten Nielsen and Claus Lundegaard
# BMC Structural Biology 2009, 9:51 doi:10.1186/1472-6807-9-51
#
# Column 1: Class assignment - B for buried or E for Exposed - Threshold: 25% exposure, but not based on RSA
# Column 2: Amino acid
# Column 3: Sequence name
# Column 4: Amino acid number
# Column 5: Relative Surface Accessibility - RSA
# Column 6: Absolute Surface Accessibility
# Column 7: Z-fit score for RSA prediction
# Column 8: Probability for Alpha-Helix
# Column 9: Probability for Beta-strand
# Column 10: Probability for Coil
E A  4OFYD                  1    0.679  74.870  -1.369   0.003   0.003   0.994
E D  4OFYD                  2    0.538  77.569   0.404   0.005   0.015   0.979
B P  4OFYD                  3    0.189  26.791  -1.503   0.020   0.205   0.775
E F  4OFYD                  4    0.508 102.036   1.344   0.003   0.718   0.279
B F  4OFYD                  5    0.146  29.242  -0.340   0.001   0.900   0.099
E L  4OFYD                  6    0.348  63.646   0.657   0.002   0.816   0.182
E E  4OFYD                  7    0.460  80.397   0.702   0.003   0.718   0.279
E S  4OFYD                  8    0.484  56.713   1.013   0.005   0.336   0.660
E P  4OFYD                  9    0.265  37.618  -1.003   0.004   0.085   0.910
E S  4OFYD                 10    0.627  73.496   0.473   0.004   0.197   0.799
E N  4OFYD                 11    0.603  88.235  -0.023   0.004   0.514   0.481
B L  4OFYD                 12    0.160  29.314   1.177   0.002   0.816   0.182
E S  4OFYD                 13    0.494  57.838   1.124   0.001   0.900   0.099
B T  4OFYD                 14    0.063   8.710   0.185   0.001   0.959   0.040
E I  4OFYD                 15    0.445  82.325   1.278   0.002   0.816   0.182
E A  4OFYD                 16    0.422  46.537   0.633   0.005   0.262   0.733
E G  4OFYD                 17    0.740  58.222  -0.255   0.005   0.015   0.979
E E  4OFYD                 18    0.436  76.099   1.063   0.004   0.085   0.910
E S  4OFYD                 19    0.524  61.378   1.317   0.004   0.616   0.381
B I  4OFYD                 20    0.033   6.123   0.094   0.001   0.900   0.099
E T  4OFYD                 21    0.369  51.236   1.945   0.001   0.959   0.040
B F  4OFYD                 22    0.036   7.205   0.570   0.001   0.959   0.040
E R  4OFYD                 23    0.343  78.501   1.952   0.001   0.900   0.099
B C  4OFYD                 24    0.016   2.289   1.214   0.001   0.900   0.099
E S  4OFYD                 25    0.404  47.384   1.654   0.001   0.959   0.040
B A  4OFYD                 26    0.051   5.587  -0.655   0.001   0.900   0.099
E E  4OFYD                 27    0.523  91.316   0.702   0.003   0.718   0.279
E K  4OFYD                 28    0.487 100.155  -1.693   0.005   0.336   0.660
E S  4OFYD                 29    0.473  55.412  -0.402   0.004   0.085   0.910
E P  4OFYD                 30    0.542  76.896  -1.446   0.005   0.015   0.979
E E  4OFYD                 31    0.543  94.775  -0.266   0.005   0.045   0.951
B P  4OFYD                 32    0.137  19.469  -1.048   0.004   0.197   0.799
E I  4OFYD                 33    0.431  79.661   0.836   0.003   0.718   0.279
B V  4OFYD                 34    0.056   8.607   0.210   0.001   0.900   0.099
B Y  4OFYD                 35    0.191  40.881   0.749   0.001   0.959   0.040
B S  4OFYD                 36    0.050   5.872   0.701   0.001   0.959   0.040
B Q  4OFYD                 37    0.164  29.344   0.851   0.001   0.959   0.040
B W  4OFYD                 38    0.036   8.610  -0.074   0.001   0.959   0.040
B K  4OFYD                 39    0.232  47.661   0.772   0.001   0.900   0.099
E S  4OFYD                 40    0.253  29.605   0.422   0.003   0.718   0.279
E N  4OFYD                 41    0.606  88.792  -0.664   0.005   0.015   0.979
E T  4OFYD                 42    0.668  92.665  -2.195   0.005   0.015   0.979
E G  4OFYD                 43    0.546  42.939  -0.162   0.004   0.616   0.381
E S  4OFYD                 44    0.487  57.065   0.503   0.003   0.718   0.279
B L  4OFYD                 45    0.097  17.742   0.077   0.023   0.655   0.322
E L  4OFYD                 46    0.389  71.208  -0.148   0.022   0.552   0.426
E G  4OFYD                 47    0.412  32.393  -1.503   0.021   0.279   0.699
E Y  4OFYD                 48    0.333  71.098  -1.505   0.019   0.141   0.840
E H  4OFYD                 49    0.558 101.409  -1.862   0.019   0.141   0.840
E Q  4OFYD                 50    0.524  93.658  -1.187   0.020   0.205   0.775
E E  4OFYD                 51    0.411  71.784  -1.003   0.021   0.451   0.528
E G  4OFYD                 52    0.415  32.637  -0.814   0.023   0.655   0.322
B I  4OFYD                 53    0.244  45.048  -1.142   0.021   0.756   0.223
E L  4OFYD                 54    0.305  55.882  -0.618   0.023   0.655   0.322
E P  4OFYD                 55    0.321  45.564  -1.506   0.022   0.552   0.426
E G  4OFYD                 56    0.420  33.023  -1.392   0.022   0.359   0.619
E H  4OFYD                 57    0.489  88.949  -1.754   0.020   0.205   0.775
E Q  4OFYD                 58    0.591 105.624  -2.171   0.004   0.085   0.910
E G  4OFYD                 59    0.384  30.189  -1.824   0.004   0.197   0.799
E R  4OFYD                 60    0.272  62.311  -0.845   0.004   0.616   0.381
B F  4OFYD                 61    0.127  25.589  -0.757   0.001   0.900   0.099
B S  4OFYD                 62    0.217  25.432  -0.490   0.001   0.900   0.099
B Y  4OFYD                 63    0.159  34.064  -0.896   0.001   0.959   0.040
B I  4OFYD                 64    0.186  34.392  -1.086   0.001   0.900   0.099
E K  4OFYD                 65    0.350  72.077  -1.056   0.002   0.816   0.182
E Q  4OFYD                 66    0.408  72.833  -1.478   0.004   0.616   0.381
E N  4OFYD                 67    0.545  79.715  -1.512   0.004   0.197   0.799
E A  4OFYD                 68    0.658  72.512  -2.081   0.004   0.085   0.910
E E  4OFYD                 69    0.622 108.594  -1.226   0.004   0.138   0.858
E E  4OFYD                 70    0.463  80.799  -0.144   0.005   0.336   0.660
B L  4OFYD                 71    0.091  16.625  -0.066   0.003   0.718   0.279
B H  4OFYD                 72    0.217  39.545   0.697   0.001   0.900   0.099
B L  4OFYD                 73    0.022   3.955   0.864   0.001   0.959   0.040
E K  4OFYD                 74    0.237  48.792   1.374   0.001   0.959   0.040
B I  4OFYD                 75    0.025   4.532   0.642   0.001   0.900   0.099
E T  4OFYD                 76    0.428  59.391   1.312   0.003   0.718   0.279
E H  4OFYD                 77    0.513  93.387   0.486   0.004   0.420   0.576
B V  4OFYD                 78    0.108  16.661   0.218   0.004   0.197   0.799
E N  4OFYD                 79    0.485  70.931   0.812   0.005   0.262   0.733
E L  4OFYD                 80    0.558 102.133   0.036   0.005   0.262   0.733
E D  4OFYD                 81    0.663  95.567   0.037   0.004   0.085   0.910
B D  4OFYD                 82    0.186  26.745  -1.139   0.004   0.085   0.910
E D  4OFYD                 83    0.534  76.906  -0.156   0.004   0.138   0.858
B G  4OFYD                 84    0.144  11.356  -1.273   0.003   0.718   0.279
E E  4OFYD                 85    0.383  66.928   2.183   0.001   0.959   0.040
B Y  4OFYD                 86    0.032   6.924   0.545   0.001   0.959   0.040
B E  4OFYD                 87    0.208  36.390   2.191   0.001   0.959   0.040
B C  4OFYD                 88    0.012   1.643   1.739   0.001   0.959   0.040
B Q  4OFYD                 89    0.166  29.719   2.019   0.001   0.959   0.040
B M  4OFYD                 90    0.018   3.602   0.893   0.001   0.959   0.040
E L  4OFYD                 91    0.302  55.260   1.150   0.001   0.959   0.040
B H  4OFYD                 92    0.115  20.919   0.110   0.001   0.900   0.099
E P  4OFYD                 93    0.499  70.865  -0.182   0.004   0.138   0.858
E E  4OFYD                 94    0.690 120.613  -0.766   0.004   0.085   0.910
E E  4OFYD                 95    0.615 107.388  -1.043   0.004   0.138   0.858
E G  4OFYD                 96    0.486  38.288  -0.821   0.005   0.262   0.733
E P  4OFYD                 97    0.515  73.050   0.138   0.003   0.718   0.279
E I  4OFYD                 98    0.286  52.892   0.656   0.001   0.900   0.099
E R  4OFYD                 99    0.372  85.096  -0.212   0.001   0.900   0.099
B A  4OFYD                100    0.219  24.134   0.730   0.001   0.900   0.099
E K  4OFYD                101    0.404  83.185   1.095   0.001   0.959   0.040
B S  4OFYD                102    0.033   3.926  -0.091   0.001   0.959   0.040
E F  4OFYD                103    0.312  62.618   1.673   0.001   0.959   0.040
B L  4OFYD                104    0.022   4.047   1.105   0.001   0.959   0.040
E N  4OFYD                105    0.249  36.468   1.944   0.001   0.959   0.040
B I  4OFYD                106    0.024   4.403   0.963   0.001   0.959   0.040
E I  4OFYD                107    0.266  49.173   0.975   0.001   0.900   0.099
B V  4OFYD                108    0.176  27.005   0.186   0.004   0.616   0.381
B P  4OFYD                109    0.231  32.821   0.722   0.004   0.138   0.858
B P  4OFYD                110    0.269  38.143  -0.839   0.018   0.019   0.964
E Q  4OFYD                111    0.588 105.017  -0.752   0.018   0.088   0.893
E L  4OFYD                112    0.343  62.767  -0.493   0.019   0.141   0.840
B V  4OFYD                113    0.180  27.727  -1.183   0.020   0.205   0.775
E Y  4OFYD                114    0.577 123.219  -1.045   0.021   0.279   0.699
B F  4OFYD                115    0.229  45.980  -0.676   0.021   0.279   0.699
E S  4OFYD                116    0.450  52.693  -1.076   0.021   0.279   0.699
E N  4OFYD                117    0.496  72.614  -1.159   0.020   0.205   0.775
E Y  4OFYD                118    0.494 105.461  -1.133   0.019   0.141   0.840
E Q  4OFYD                119    0.573 102.338  -1.040   0.018   0.088   0.893
E P  4OFYD                120    0.498  70.680  -1.244   0.018   0.047   0.935
E N  4OFYD                121    0.502  73.551  -1.564   0.018   0.047   0.935
E S  4OFYD                122    0.519  60.874  -0.717   0.004   0.197   0.799
E I  4OFYD                123    0.530  98.013  -0.486   0.004   0.616   0.381
B I  4OFYD                124    0.201  37.130   0.633   0.001   0.900   0.099
E A  4OFYD                125    0.417  45.942   0.790   0.001   0.959   0.040
B V  4OFYD                126    0.097  14.863   0.335   0.001   0.959   0.040
E K  4OFYD                127    0.438  90.035   0.785   0.001   0.900   0.099
E E  4OFYD                128    0.469  82.004   0.171   0.005   0.336   0.660
E N  4OFYD                129    0.697 101.968  -0.795   0.005   0.015   0.979
E T  4OFYD                130    0.473  65.647   1.018   0.004   0.085   0.910
E P  4OFYD                131    0.535  75.902   1.379   0.004   0.616   0.381
B L  4OFYD                132    0.047   8.624   0.803   0.001   0.900   0.099
E N  4OFYD                133    0.388  56.759   2.117   0.001   0.959   0.040
B I  4OFYD                134    0.034   6.234   0.490   0.001   0.959   0.040
E T  4OFYD                135    0.343  47.505   1.687   0.001   0.959   0.040
B C  4OFYD                136    0.016   2.246   1.122   0.011   0.918   0.071
E V  4OFYD                137    0.435  66.890   1.605   0.001   0.900   0.099
B V  4OFYD                138    0.055   8.392  -0.347   0.002   0.816   0.182
E P  4OFYD                139    0.500  70.893   0.252   0.004   0.420   0.576
E N  4OFYD                140    0.711 104.032  -2.125   0.005   0.045   0.951
E V  4OFYD                141    0.515  79.155  -1.229   0.005   0.045   0.951
E K  4OFYD                142    0.539 110.913  -0.607   0.005   0.045   0.951
B P  4OFYD                143    0.207  29.302  -0.623   0.004   0.138   0.858
E E  4OFYD                144    0.499  87.228   0.806   0.005   0.262   0.733
B P  4OFYD                145    0.065   9.266  -0.405   0.004   0.616   0.381
E E  4OFYD                146    0.333  58.175   1.201   0.001   0.900   0.099
B V  4OFYD                147    0.037   5.702   0.343   0.001   0.959   0.040
B L  4OFYD                148    0.228  41.692   1.775   0.001   0.959   0.040
B W  4OFYD                149    0.034   8.201   0.235   0.006   0.962   0.032
B Y  4OFYD                150    0.243  51.950   0.950   0.001   0.959   0.040
B M  4OFYD                151    0.216  43.282   1.033   0.002   0.816   0.182
E D  4OFYD                152    0.629  90.567  -0.158   0.005   0.015   0.979
E G  4OFYD                153    0.737  57.978  -1.528   0.016   0.005   0.979
E K  4OFYD                154    0.570 117.249   0.221   0.003   0.718   0.279
E V  4OFYD                155    0.487  74.913   0.548   0.003   0.718   0.279
B M  4OFYD                156    0.087  17.429   0.081   0.022   0.552   0.426
E S  4OFYD                157    0.492  57.627   0.261   0.022   0.359   0.619
E R  4OFYD                158    0.496 113.607  -1.437   0.019   0.141   0.840
E D  4OFYD                159    0.575  82.886  -1.944   0.018   0.047   0.935
E V  4OFYD                160    0.529  81.277  -1.930   0.018   0.088   0.893
E K  4OFYD                161    0.559 114.883  -0.606   0.021   0.451   0.528
E Q  4OFYD                162    0.356  63.599  -0.555   0.023   0.655   0.322
E A  4OFYD                163    0.368  40.532  -0.925   0.021   0.756   0.223
E S  4OFYD                164    0.391  45.849  -1.085   0.021   0.756   0.223
E T  4OFYD                165    0.355  49.211  -0.595   0.021   0.756   0.223
E P  4OFYD                166    0.390  55.341  -1.216   0.023   0.655   0.322
E H  4OFYD                167    0.501  91.114  -1.517   0.004   0.514   0.481
E L  4OFYD                168    0.536  98.142  -2.370   0.005   0.262   0.733
E N  4OFYD                169    0.584  85.512  -1.671   0.005   0.262   0.733
E K  4OFYD                170    0.322  66.194  -1.297   0.004   0.514   0.481
E T  4OFYD                171    0.435  60.334  -1.114   0.003   0.718   0.279
B F  4OFYD                172    0.246  49.312  -0.980   0.002   0.816   0.182
E T  4OFYD                173    0.353  48.947  -0.918   0.004   0.616   0.381
E V  4OFYD                174    0.364  55.978  -1.420   0.004   0.514   0.481
E Y  4OFYD                175    0.430  91.934  -0.971   0.004   0.514   0.481
B T  4OFYD                176    0.107  14.883  -1.264   0.003   0.718   0.279
B S  4OFYD                177    0.212  24.870   0.435   0.001   0.900   0.099
B L  4OFYD                178    0.026   4.742   0.483   0.001   0.900   0.099
E V  4OFYD                179    0.253  38.917   1.347   0.001   0.959   0.040
B V  4OFYD                180    0.041   6.317  -0.258   0.001   0.900   0.099
E Q  4OFYD                181    0.409  73.047   1.138   0.002   0.816   0.182
E S  4OFYD                182    0.531  62.257  -0.016   0.004   0.616   0.381
B D  4OFYD                183    0.227  32.667   0.165   0.005   0.336   0.660
E R  4OFYD                184    0.507 116.103   0.435   0.005   0.262   0.733
E N  4OFYD                185    0.599  87.664   0.022   0.004   0.197   0.799
E D  4OFYD                186    0.664  95.740  -0.474   0.004   0.085   0.910
B H  4OFYD                187    0.263  47.894  -1.012   0.005   0.045   0.951
E G  4OFYD                188    0.526  41.365  -0.481   0.004   0.138   0.858
B K  4OFYD                189    0.137  28.119  -0.692   0.004   0.616   0.381
E V  4OFYD                190    0.372  57.161   1.833   0.001   0.900   0.099
B I  4OFYD                191    0.038   6.956   0.702   0.001   0.959   0.040
B T  4OFYD                192    0.214  29.682   1.883   0.001   0.959   0.040
B C  4OFYD                193    0.012   1.629   1.687   0.001   0.959   0.040
B E  4OFYD                194    0.212  37.036   1.721   0.001   0.959   0.040
B A  4OFYD                195    0.025   2.788   0.358   0.001   0.959   0.040
E F  4OFYD                196    0.388  77.892   1.099   0.001   0.959   0.040
B Q  4OFYD                197    0.112  19.985  -0.471   0.004   0.616   0.381
E K  4OFYD                198    0.583 119.882   0.019   0.004   0.138   0.858
E E  4OFYD                199    0.426  74.492  -1.091   0.004   0.138   0.858
E T  4OFYD                200    0.454  62.998  -1.118   0.004   0.197   0.799
E D  4OFYD                201    0.626  90.149  -0.686   0.005   0.262   0.733
E I  4OFYD                202    0.488  90.317  -0.567   0.004   0.420   0.576
E R  4OFYD                203    0.529 121.233  -0.454   0.003   0.718   0.279
E I  4OFYD                204    0.282  52.170   0.075   0.002   0.816   0.182
E T  4OFYD                205    0.450  62.387  -0.379   0.002   0.816   0.182
B T  4OFYD                206    0.241  33.468   0.271   0.001   0.900   0.099
E N  4OFYD                207    0.471  68.998   0.947   0.001   0.900   0.099
B T  4OFYD                208    0.042   5.770  -0.043   0.001   0.959   0.040
E T  4OFYD                209    0.311  43.136   1.635   0.001   0.959   0.040
B L  4OFYD                210    0.027   4.980   0.565   0.001   0.959   0.040
E D  4OFYD                211    0.318  45.809   1.435   0.001   0.959   0.040
B V  4OFYD                212    0.044   6.778  -0.640   0.001   0.900   0.099
E L  4OFYD                213    0.410  74.998   0.455   0.001   0.900   0.099
B F  4OFYD                214    0.219  43.993   0.041   0.004   0.514   0.481
E P  4OFYD                215    0.364  51.723   0.240   0.004   0.085   0.910
E P  4OFYD                216    0.432  61.315  -1.351   0.005   0.045   0.951
E S  4OFYD                217    0.596  69.816  -0.892   0.004   0.138   0.858
E D  4OFYD                218    0.438  63.058  -0.686   0.004   0.197   0.799
B P  4OFYD                219    0.201  28.508  -0.576   0.004   0.514   0.481
E T  4OFYD                220    0.421  58.420   0.422   0.002   0.816   0.182
B V  4OFYD                221    0.060   9.283  -0.842   0.001   0.900   0.099
E E  4OFYD                222    0.384  67.050   0.043   0.001   0.900   0.099
B I  4OFYD                223    0.252  46.712  -0.909   0.003   0.718   0.279
E L  4OFYD                224    0.375  68.699  -0.477   0.005   0.336   0.660
E R  4OFYD                225    0.490 112.279  -1.768   0.005   0.045   0.951
E N  4OFYD                226    0.651  95.321  -0.612   0.004   0.085   0.910
E P  4OFYD                227    0.629  89.241  -1.408   0.005   0.262   0.733
B S  4OFYD                228    0.251  29.382   0.293   0.004   0.616   0.381
E A  4OFYD                229    0.471  51.893   0.558   0.002   0.816   0.182
B L  4OFYD                230    0.075  13.696  -0.281   0.002   0.816   0.182
E R  4OFYD                231    0.453 103.760   0.795   0.003   0.718   0.279
E S  4OFYD                232    0.486  56.983   0.238   0.005   0.262   0.733
E G  4OFYD                233    0.691  54.342  -1.006   0.005   0.045   0.951
E D  4OFYD                234    0.420  60.493   0.780   0.004   0.085   0.910
E N  4OFYD                235    0.538  78.705   1.508   0.003   0.718   0.279
B V  4OFYD                236    0.042   6.502   0.194   0.001   0.959   0.040
E T  4OFYD                237    0.364  50.417   1.904   0.001   0.959   0.040
B I  4OFYD                238    0.036   6.660   0.488   0.001   0.959   0.040
E A  4OFYD                239    0.323  35.551   1.773   0.001   0.959   0.040
B C  4OFYD                240    0.017   2.387   1.171   0.001   0.959   0.040
E S  4OFYD                241    0.401  46.997   1.644   0.001   0.959   0.040
B V  4OFYD                242    0.064   9.883  -0.541   0.001   0.959   0.040
E T  4OFYD                243    0.464  64.357   0.040   0.004   0.616   0.381
E G  4OFYD                244    0.662  52.115  -2.046   0.004   0.138   0.858
E G  4OFYD                245    0.546  42.954  -1.754   0.005   0.045   0.951
E N  4OFYD                246    0.504  73.756  -0.481   0.005   0.045   0.951
B P  4OFYD                247    0.192  27.231  -0.829   0.004   0.138   0.858
E P  4OFYD                248    0.443  62.848   0.669   0.005   0.262   0.733
B P  4OFYD                249    0.071  10.132  -0.767   0.004   0.514   0.481
E D  4OFYD                250    0.377  54.383   0.884   0.001   0.900   0.099
B V  4OFYD                251    0.039   6.025   0.200   0.001   0.959   0.040
E F  4OFYD                252    0.287  57.501   1.831   0.001   0.959   0.040
B W  4OFYD                253    0.029   6.878   0.438   0.001   0.959   0.040
E Y  4OFYD                254    0.250  53.468   1.298   0.001   0.959   0.040
B H  4OFYD                255    0.215  39.145   1.005   0.002   0.816   0.182
E E  4OFYD                256    0.600 104.768  -0.429   0.018   0.019   0.964
E N  4OFYD                257    0.770 112.728  -1.455   0.018   0.019   0.964
E K  4OFYD                258    0.577 118.586   0.341   0.004   0.616   0.381
E R  4OFYD                259    0.550 125.996   0.741   0.003   0.718   0.279
B L  4OFYD                260    0.072  13.275  -0.001   0.022   0.552   0.426
E Q  4OFYD                261    0.492  87.943   0.728   0.005   0.262   0.733
E S  4OFYD                262    0.605  70.929  -1.270   0.004   0.085   0.910
E H  4OFYD                263    0.608 110.522  -2.364   0.005   0.045   0.951
E S  4OFYD                264    0.593  69.464  -2.110   0.004   0.138   0.858
E T  4OFYD                265    0.507  70.307  -0.143   0.004   0.616   0.381
B L  4OFYD                266    0.241  44.145  -0.416   0.002   0.816   0.182
E D  4OFYD                267    0.437  62.928  -0.388   0.001   0.900   0.099
B T  4OFYD                268    0.289  40.057  -1.181   0.001   0.900   0.099
E R  4OFYD                269    0.421  96.455  -0.650   0.001   0.900   0.099
E S  4OFYD                270    0.324  38.008  -1.588   0.001   0.900   0.099
E K  4OFYD                271    0.513 105.462  -0.935   0.003   0.718   0.279
E E  4OFYD                272    0.507  88.590  -1.849   0.005   0.336   0.660
E I  4OFYD                273    0.493  91.186  -1.284   0.005   0.262   0.733
E K  4OFYD                274    0.457  94.005  -0.522   0.005   0.336   0.660
B N  4OFYD                275    0.109  15.899  -0.514   0.004   0.616   0.381
B I  4OFYD                276    0.179  33.078   0.257   0.001   0.900   0.099
B Y  4OFYD                277    0.023   4.894   0.858   0.001   0.959   0.040
B S  4OFYD                278    0.204  23.874   1.448   0.001   0.959   0.040
B F  4OFYD                279    0.027   5.339   0.726   0.001   0.959   0.040
E I  4OFYD                280    0.402  74.333   1.523   0.002   0.816   0.182
E A  4OFYD                281    0.462  50.879   0.412   0.004   0.616   0.381
B S  4OFYD                282    0.085   9.997   0.099   0.005   0.262   0.733
E Q  4OFYD                283    0.404  72.083   0.956   0.004   0.197   0.799
E N  4OFYD                284    0.608  88.982   0.327   0.004   0.197   0.799
E D  4OFYD                285    0.663  95.610  -0.009   0.004   0.085   0.910
B N  4OFYD                286    0.215  31.476  -1.190   0.005   0.045   0.951
E M  4OFYD                287    0.508 101.711  -0.591   0.004   0.138   0.858
B A  4OFYD                288    0.191  21.059  -1.271   0.004   0.616   0.381
E E  4OFYD                289    0.446  77.934   1.790   0.001   0.900   0.099
B Y  4OFYD                290    0.047  10.023   0.658   0.001   0.959   0.040
E E  4OFYD                291    0.289  50.558   2.089   0.001   0.959   0.040
B C  4OFYD                292    0.014   1.909   1.534   0.001   0.959   0.040
E R  4OFYD                293    0.257  58.922   2.165   0.001   0.959   0.040
B A  4OFYD                294    0.028   3.064  -0.111   0.001   0.959   0.040
E N  4OFYD                295    0.395  57.799   0.702   0.001   0.959   0.040
B N  4OFYD                296    0.077  11.214  -0.840   0.004   0.616   0.381
E S  4OFYD                297    0.614  71.949  -0.125   0.004   0.138   0.858
E R  4OFYD                298    0.391  89.562  -0.979   0.004   0.197   0.799
E T  4OFYD                299    0.423  58.628  -1.284   0.005   0.262   0.733
E G  4OFYD                300    0.587  46.205  -0.962   0.004   0.197   0.799
E N  4OFYD                301    0.522  76.392  -0.888   0.005   0.336   0.660
E P  4OFYD                302    0.516  73.206  -0.676   0.003   0.718   0.279
E K  4OFYD                303    0.271  55.786  -0.064   0.001   0.900   0.099
E R  4OFYD                304    0.465 106.439   0.027   0.001   0.900   0.099
B K  4OFYD                305    0.220  45.357   0.614   0.001   0.959   0.040
E A  4OFYD                306    0.392  43.231   0.651   0.001   0.959   0.040
B M  4OFYD                307    0.053  10.705   0.226   0.001   0.959   0.040
E K  4OFYD                308    0.315  64.837   1.697   0.001   0.959   0.040
B L  4OFYD                309    0.021   3.918   1.192   0.001   0.959   0.040
E E  4OFYD                310    0.281  49.161   1.720   0.001   0.959   0.040
B V  4OFYD                311    0.041   6.348  -0.092   0.001   0.900   0.099
E N  4OFYD                312    0.381  55.837   0.769   0.002   0.816   0.182
B Y  4OFYD                313    0.281  59.943   0.045   0.004   0.420   0.576
E P  4OFYD                314    0.292  41.435   0.400   0.004   0.085   0.910
B P  4OFYD                315    0.108  15.254  -1.177   0.020   0.205   0.775
E A  4OFYD                316    0.442  48.741   0.899   0.002   0.816   0.182
B S  4OFYD                317    0.173  20.299  -0.149   0.001   0.900   0.099
B V  4OFYD                318    0.244  37.580   0.129   0.018   0.846   0.136
E E  4OFYD                319    0.432  75.401  -0.141   0.023   0.655   0.322
E L  4OFYD                320    0.460  84.226   0.470   0.005   0.336   0.660
E F  4OFYD                321    0.295  59.287  -1.096   0.004   0.085   0.910
E G  4OFYD                322    0.610  48.015  -0.432   0.004   0.085   0.910
E E  4OFYD                323    0.648 113.188  -0.724   0.020   0.205   0.775
E S  4OFYD                324    0.351  41.172   0.109   0.004   0.514   0.481
E N  4OFYD                325    0.566  82.921   0.187   0.003   0.718   0.279
B I  4OFYD                326    0.099  18.296   0.290   0.003   0.718   0.279
E R  4OFYD                327    0.504 115.302   0.517   0.004   0.616   0.381
E Y  4OFYD                328    0.466  99.520   0.284   0.005   0.336   0.660
E G  4OFYD                329    0.652  51.328  -0.976   0.005   0.045   0.951
E S  4OFYD                330    0.443  51.908   0.450   0.004   0.085   0.910
E S  4OFYD                331    0.502  58.823   1.042   0.003   0.718   0.279
B A  4OFYD                332    0.041   4.540   0.517   0.001   0.959   0.040
E N  4OFYD                333    0.345  50.493   1.817   0.001   0.959   0.040
B I  4OFYD                334    0.034   6.272   0.762   0.001   0.959   0.040
E Q  4OFYD                335    0.297  53.026   1.553   0.001   0.959   0.040
B C  4OFYD                336    0.020   2.794   1.092   0.001   0.959   0.040
E K  4OFYD                337    0.391  80.346   1.898   0.001   0.959   0.040
B S  4OFYD                338    0.054   6.387  -0.488   0.001   0.959   0.040
E L  4OFYD                339    0.476  87.137   0.515   0.003   0.718   0.279
E P  4OFYD                340    0.673  95.485  -1.275   0.004   0.197   0.799
E S  4OFYD                341    0.589  68.996  -1.894   0.005   0.045   0.951
E N  4OFYD                342    0.544  79.700  -0.175   0.005   0.015   0.979
B P  4OFYD                343    0.186  26.450  -0.558   0.004   0.138   0.858
E A  4OFYD                344    0.423  46.571   0.917   0.005   0.262   0.733
B S  4OFYD                345    0.085   9.939  -0.056   0.004   0.616   0.381
E Q  4OFYD                346    0.328  58.634   0.972   0.001   0.900   0.099
B I  4OFYD                347    0.030   5.606   0.561   0.001   0.959   0.040
B T  4OFYD                348    0.227  31.513   2.001   0.001   0.959   0.040
B W  4OFYD                349    0.027   6.421   0.662   0.001   0.959   0.040
B I  4OFYD                350    0.216  39.868   1.392   0.001   0.959   0.040
B I  4OFYD                351    0.182  33.689   1.234   0.001   0.900   0.099
E N  4OFYD                352    0.568  83.126   0.234   0.005   0.045   0.951
E G  4OFYD                353    0.737  58.033  -1.502   0.018   0.019   0.964
E R  4OFYD                354    0.599 137.148   0.267   0.003   0.718   0.279
E S  4OFYD                355    0.520  60.991   0.672   0.003   0.718   0.279
B V  4OFYD                356    0.085  13.111   0.027   0.021   0.451   0.528
E P  4OFYD                357    0.525  74.526   0.093   0.021   0.279   0.699
E T  4OFYD                358    0.514  71.333  -0.818   0.004   0.138   0.858
E P  4OFYD                359    0.594  84.331  -2.418   0.005   0.015   0.979
E T  4OFYD                360    0.655  90.835  -2.516   0.005   0.045   0.951
E Q  4OFYD                361    0.573 102.302  -0.814   0.005   0.336   0.660
E R  4OFYD                362    0.347  79.577  -0.349   0.004   0.616   0.381
E E  4OFYD                363    0.320  55.904  -0.812   0.001   0.900   0.099
B F  4OFYD                364    0.235  47.245  -0.664   0.001   0.959   0.040
E V  4OFYD                365    0.327  50.321  -0.370   0.001   0.959   0.040
B V  4OFYD                366    0.251  38.502  -1.006   0.001   0.900   0.099
E E  4OFYD                367    0.577 100.854  -1.350   0.004   0.514   0.481
E N  4OFYD                368    0.731 106.989  -1.774   0.005   0.045   0.951
E G  4OFYD                369    0.487  38.303  -1.845   0.004   0.085   0.910
E I  4OFYD                370    0.470  86.950  -0.954   0.003   0.718   0.279
B V  4OFYD                371    0.137  21.118  -0.115   0.001   0.900   0.099
E S  4OFYD                372    0.288  33.730  -0.207   0.001   0.900   0.099
B S  4OFYD                373    0.122  14.252  -0.879   0.001   0.900   0.099
E S  4OFYD                374    0.321  37.598  -0.827   0.003   0.718   0.279
E C  4OFYD                375    0.365  51.218  -1.101   0.004   0.616   0.381
B V  4OFYD                376    0.163  25.038  -1.358   0.023   0.655   0.322
E S  4OFYD                377    0.287  33.625  -0.991   0.002   0.816   0.182
B V  4OFYD                378    0.205  31.585  -0.498   0.021   0.756   0.223
B H  4OFYD                379    0.237  43.056  -0.885   0.023   0.655   0.322
E S  4OFYD                380    0.335  39.227  -0.754   0.023   0.655   0.322
B N  4OFYD                381    0.188  27.538  -1.079   0.023   0.655   0.322
E E  4OFYD                382    0.494  86.389  -0.458   0.022   0.552   0.426
E L  4OFYD                383    0.535  97.904  -1.031   0.021   0.451   0.528
B S  4OFYD                384    0.278  32.523  -0.596   0.022   0.359   0.619
E V  4OFYD                385    0.474  72.854  -0.387   0.005   0.262   0.733
E E  4OFYD                386    0.571  99.824  -0.462   0.004   0.197   0.799
E A  4OFYD                387    0.612  67.420  -0.642   0.004   0.138   0.858
B H  4OFYD                388    0.143  26.030  -1.783   0.004   0.138   0.858
E Q  4OFYD                389    0.348  62.224  -0.939   0.005   0.262   0.733
B I  4OFYD                390    0.100  18.426  -2.153   0.003   0.718   0.279
E N  4OFYD                391    0.371  54.373   1.622   0.001   0.900   0.099
B V  4OFYD                392    0.036   5.487   0.453   0.001   0.959   0.040
B E  4OFYD                393    0.230  40.251   1.982   0.001   0.959   0.040
B C  4OFYD                394    0.014   1.909   1.573   0.001   0.959   0.040
E M  4OFYD                395    0.248  49.645   2.012   0.001   0.959   0.040
B A  4OFYD                396    0.031   3.460  -0.368   0.001   0.959   0.040
E T  4OFYD                397    0.392  54.329   0.920   0.001   0.959   0.040
B N  4OFYD                398    0.095  13.849   0.194   0.003   0.718   0.279
E P  4OFYD                399    0.620  88.021   0.295   0.004   0.138   0.858
E E  4OFYD                400    0.403  70.474  -0.536   0.005   0.336   0.660
E G  4OFYD                401    0.430  33.857  -1.368   0.004   0.616   0.381
E S  4OFYD                402    0.493  57.733  -0.309   0.003   0.718   0.279
E S  4OFYD                403    0.313  36.648  -0.691   0.001   0.900   0.099
E A  4OFYD                404    0.474  52.279   0.025   0.001   0.900   0.099
B K  4OFYD                405    0.194  39.844   0.459   0.001   0.959   0.040
E Q  4OFYD                406    0.405  72.351   0.577   0.001   0.959   0.040
B H  4OFYD                407    0.035   6.439  -0.286   0.001   0.959   0.040
E V  4OFYD                408    0.309  47.432   1.960   0.001   0.959   0.040
B I  4OFYD                409    0.024   4.385   0.877   0.001   0.959   0.040
E K  4OFYD                410    0.291  59.920   1.916   0.006   0.962   0.032
B I  4OFYD                411    0.036   6.660   0.011   0.001   0.900   0.099
E I  4OFYD                412    0.418  77.367   1.055   0.018   0.846   0.136
E A  4OFYD                413    0.316  34.812  -0.142   0.021   0.451   0.528
E P  4OFYD                414    0.388  55.071   0.110   0.018   0.047   0.935
E H  4OFYD                415    0.333  60.482  -0.809   0.005   0.045   0.951
E H  4OFYD                416    0.637 115.779   0.387   0.004   0.085   0.910
B H  4OFYD                417    0.166  30.104  -0.741   0.004   0.085   0.910
E H  4OFYD                418    0.602 109.540   0.456   0.018   0.088   0.893
E H  4OFYD                419    0.603 109.777  -0.799   0.018   0.047   0.935
E H  4OFYD                420    0.736 133.860  -2.680   0.003   0.003   0.994

Explain the output.  Go back.
```

---
